# Tenomodulin Inhibits Retinal Neovascularization in a Mouse Model of Oxygen-Induced Retinopathy

**DOI:** 10.3390/ijms131115373

**Published:** 2012-11-20

**Authors:** Wei Wang, Zhongqiu Li, Tatsuhiko Sato, Yusuke Oshima

**Affiliations:** 1 Department of Ophthalmology, Beijing Chao-Yang Hospital, Capital Medical University, Beijing 100020, China; E-Mail: lizhqiu@gmail.com; 2 Department of Ophthalmology and Visual Science, Osaka University Medical School, Yamadaoka, Suita, Osaka 5650871, Japan; E-Mails: tatsusatou@gmail.com (T.S.); oshima@ophthal.med.osaka-u.ac.jp (Y.O.)

**Keywords:** tenomodulin, retinal neovascularization, C57BL/6J mouse, proliferation, angiogenesis

## Abstract

We aimed to determine the anti-angiogenic effect of tenomodulin (TeM) on retinal neovascularization in an oxygen-induced retinopathy (OIR) mouse model. OIR was induced in C57BL/6 mice by exposing seven-day-old mice to 75% oxygen for five days followed by room air for five days. Control mice were exposed to room air from birth until postnatal day 17. Mice received intravitreal injections of 1 μg of TeM in one eye and PBS in the contralateral eye at P7 before being exposed to 75% oxygen. Eyes were collected at postnatal day 17. Retinal blood vessel patterns were visualized by fluorescein angiography. We quantified the number of neovascular nuclei that were present beyond the inner limiting membrane (ILM) using histological methods with a masked approach. Furthermore, double immunohistochemical staining of TeM was performed on retinas to identify nuclei protruding into the vitreous cavity. Western blot was used to detect exogenous TeM protein. The central nonperfusion area (NPA, mm^2^) of TeM-injected eyes was significantly different from that of OIR and PBS-injected eyes, and the number of nuclei in new blood vessels breaking through the ILM in each retinal cross-section significantly differed from that of OIR eyes and PBS-injected control eyes. Cellular nuclei of new blood vessels protruding into the vitreous cavity were also observed in TeM-injected retinas by immunohistochemistry. Western blotting revealed a 16-kDa immunoreactive protein, indicating incorporation of an exogenous TeM fragment into the retina. Our data shows that TeM can effectively inhibit pathological angiogenesis in mouse eyes; indicating its potential role in prevention and treatment of ocular neovascularization.

## 1. Introduction

Retinal neovascularization, the pathological growth of new blood vessels, is characteristic of several eye diseases that cause catastrophic vision loss throughout the world. Thus, the control of ocular neovascularization is promising for the treatment of these disorders [1,2]. However, the current ability to prevent retinal neovascularization is severely limited, and there are no preventive or early intervention treatment modalities available for these diseases [3–5]. The current therapies of laser photocoagulation and cryoablation can slow the deterioration of vision, but they also have some side effects and complications such as killing photoreceptor cells, damaging the visual field, and resulting in limited vision loss [4]. The common pathological changes of such lesions are hypoxia in retinal tissue and structural damage to the retinal microcirculation, which lead to proliferation of vascular endothelial cells and a thickening of the basement membrane followed by neovascularization [4]. It is postulated that angiogenesis results from an imbalance between angiogenic stimulators and inhibitors in a given tissue bed [6–8]. Under some pathological conditions, however, an imbalance between these factors disturbs the vascular quiescence that needs to be maintained. Numerous proangiogenic molecules have been proposed to play a role in retinal neovascularization, including vascular endothelial growth factor (VEGF), the insulin-like growth factors (IGF), basic fibroblast growth factor (bFGF), hepatocyte factor, and transforming growth factor (TGF)-β [9–13]. Ocular tissues are maintained under physiological conditions without the occurrence of neovascularization, suggesting the presence of angiogenesis inhibitors in the tissue. Thus, a balance of inducers and inhibitors of endothelial cell proliferation and angiogenesis may regulate the promotion or suppression of vascularization in the eye [14]. Little is known, however, about angiogenic inhibitory factors. The existence of angiogenic inhibitors has been predicted in retinal pigment epithelium [15], the vitreous body [16,17], and the lens [18], although most of these inhibitors have not yet been isolated.

Tenomodulin (TeM; also referred to as Tnmd or tendin), a novel member of the tumor necrosis factor family [19], has been newly identified as a transmembrane-type angiogenesis inhibitor [20]. TeM is a type II transmembrane protein that is specifically expressed in dense connective tissues having little vascularity, including tendons, ligaments, the epimysium of skeletal muscle, the cornea, and the sclera [20–24]. Mice lacking TeM display a severe decrease in tenocyte proliferation in tendons as newborns, and a disrupted collagen fibril structure in adulthood [25]. A limited number of studies have examined TeM as an inhibitor of angiogenesis, and have shown that it inhibits proliferation and tube morphogenesis of vascular endothelial cells *in vitro*, and that it suppresses tumorigenesis *in vivo*[20,26–28]. However, the role of TeM in retinal neovascularization is unclear, and its potential clinical usefulness as a modulator of neovascularization *in vivo* has not been extensively studied. In this study, we explored the anti-angiogenic potential of TeM on retinal neovascularization in a mouse model, using intravitreal administration.

## 2. Results

### 2.1. Quantification of the Central Nonperfusion Area of the Retina

**Figure 1:**
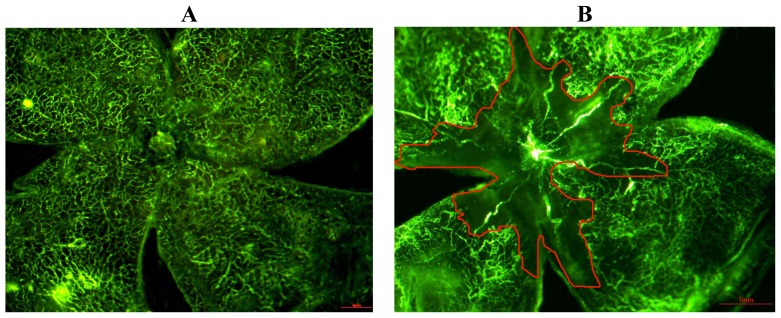

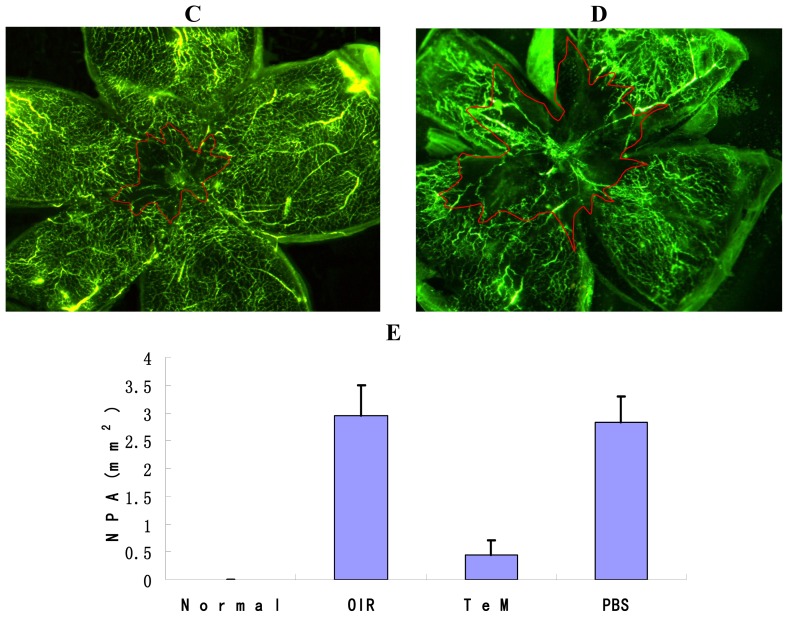
Comparison of flat-mounted fluorescein-perfused 17-day-old mouse retinas. (**A**) Both superficial and deep vascular layers of retina extend from the optic nerve to the retinal periphery in a uniformly distributed reticular pattern in the normoxic mice. No nonperfused area (NPA) was seen in the normal 17-day-old mice; (**B**) A central nonperfused area, as well as torturous and dilated vessels between the hypoperfused, and the perfuse areas were seen in the hyperoxic mice; (**C**) Fewer central nonperfused areas were seen in the TeM-injected mice; (**D**) The nonperfused area was clearly abnormal in the PBS-injected mice. The red-circled regions were mapped out by an observer, and areas were calculated using NIH ImageJ software (Scale bar: 1 mm, 40×); (**E**) A comparison of central nonperfusion areas in retina (*n* = 8), compared with OIR- and PBS-injected mice, the retinal central ischemic areas of TeM-injected mice were significantly different (*F* = 130.244, *p* < 0.01). Data shown are the mean ± SD (one-way ANOVA, Scheffé’s pairwise comparison).

The morphologic characteristics of retinal vessels were observed in retinal flat mounts after fluorescein-dextran perfusion. The retina of the P17 normoxia mouse had two layers of vessels that extended from the optic nerve to the periphery, and there was no central nonperfused area (Figure 1A). The retina of the P17 hyperoxia mouse had a prominent nonperfused area in the center, and tortuous, dilated vessels between the perfused and nonperfused area (Figure 1B). In the TeM-injected mouse (Figure 1C), the central ischemic area showed significant differences from that of the hyperoxia mouse (*p* < 0.001), and the PBS-injected contralateral control eye (Figure 1D) (*p* < 0.001). There were also very few abnormal vessels, although signs of central vaso-obliteration and neovascularization were still present (Figure 1C).

### 2.2. Histopathology

**Figure 2:**
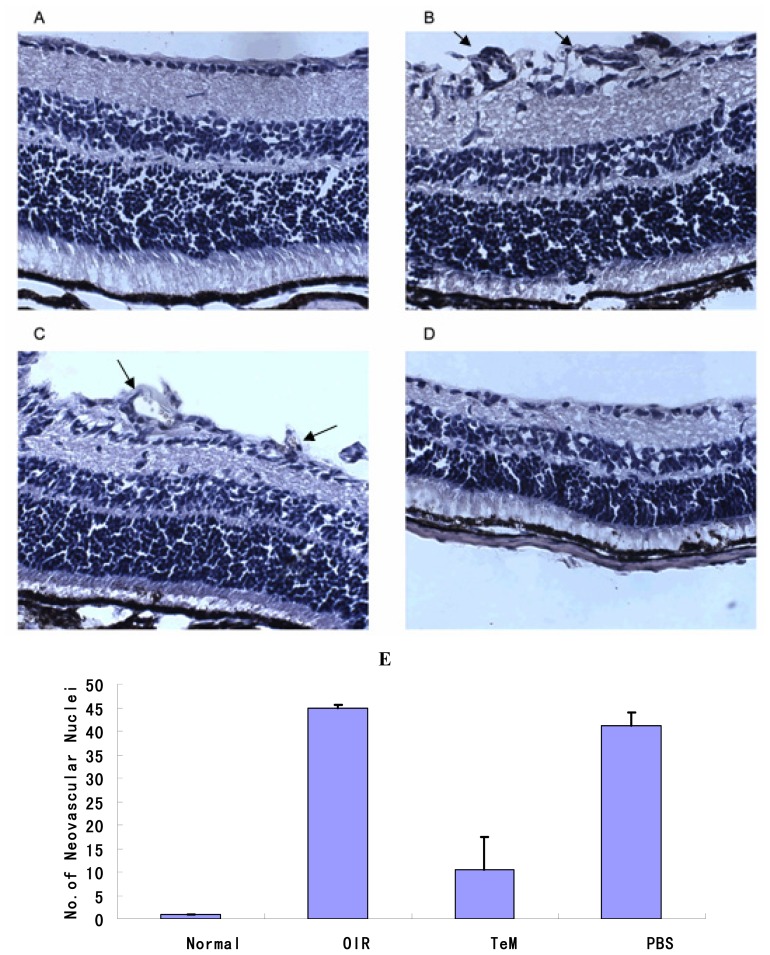
HE staining of frozen sections from 17-day-old mice (40×). (**A**) No cells were observed to be breaking through the internal limiting membrane (ILM) in the normoxic group; (**B**) Endothelial cells breaking though the ILM were seen in the oxygen-exposed group; (**C**) Endothelial cells breaking through the ILM were also found in PBS-injected eyes; (**D**) Fewer cells were seen breaking though the ILM in the TeM group. Arrows: clusters of blood vessels in retinal sections; (**E**) Graphs showing the average number of neovascular nuclei per retinal section calculated from analysis of HE staining of frozen sections from 17-day-old mice (*n* = 15). Compared with OIR mice and PBS-injected control mice, the average number of neovascular nuclei per retinal section of TeM-injected mice was significantly different (*p* < 0.01). Data shown are the mean ± SD. (one-way ANOVA, Scheffé’s pairwise comparison). The results were obtained from at least three independent experiments.

For quantitative assessment of the vasoproliferation response, HE staining was performed, and the nuclei of new blood vessels extending beyond the inner limiting membrane (ILM) into the vitreous body were counted. An average of 15–18 sections for each group was assessed. Either no neovascular nuclei or very few nuclei were found in retinas of mice raised in the normoxic condition (Figure 2A), whereas both OIR and PBS-injected mice retinas showed active neovascular responses. HE staining was observed in large clusters of blood vessels protruding from the inner retina into the vitreous cavity (Figure 2B,C). The mean numbers of neovascular nuclei counted in OIR and PBS-injected retinas were 44.93 ± 6.78 and 41.07 ± 7.31, respectively. In contrast, fewer blood vessels were observed beyond the ILM in TeM-injected mice (Figure 2D); the mean number of neovascular nuclei was 10.57 ± 2.95. There were significant differences (*p* < 0.01) in the means among TeM-injected mice and OIR mice, and TeM-injected eyes and PBS-injected control eyes. Thus, prophylactic injection of TeM significantly attenuated the neovascularization response.

### 2.3. CD31 Staining of Vascular Cells

**Figure 3:**
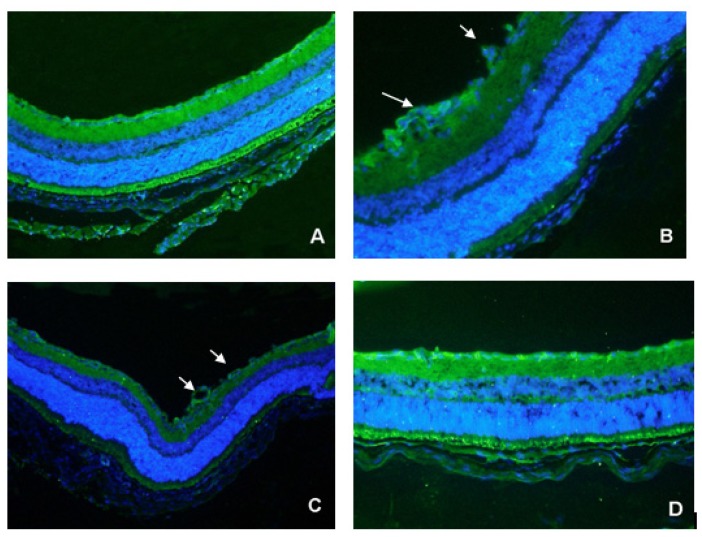
Double immunohistochemical staining of frozen retinal sections of P17 C57BL/6 mice. Serial sections were treated with the polyclonal antibody, CD31. A green signal indicates positive staining for vessels, and blue signals indicate nuclei. (**A**) Normoxia condition; (**B**) Hyperoxia condition; (**C**) Injected with PBS before being exposed to 75% oxygen; (**D**) Injected with TeM before being exposed to 75% oxygen. Arrows: new blood vessels protruding into the vitreous cavity (B, C). Magnification: 100×.

The nuclei of new blood vessels protruding from the inner retina into the vitreous cavity were observed in double immunohistochemical-stained frozen retinal sections of P17 C57BL/6 mice (Figure 3). CD31 selectively stains vascular cells. Either no neovascular nuclei, or very few nuclei were found in the retinas of mice raised in normoxic conditions (Figure 3A), whereas both OIR (Figure 3B) and PBS-injected (Figure 3C) mice retinas showed active neovascular responses. The TeM-injected retina exhibited very few neovascular nuclei (Figure 3D).

### 2.4. Detection of Exogenous TeM Protein in Retinas

**Figure 4:**
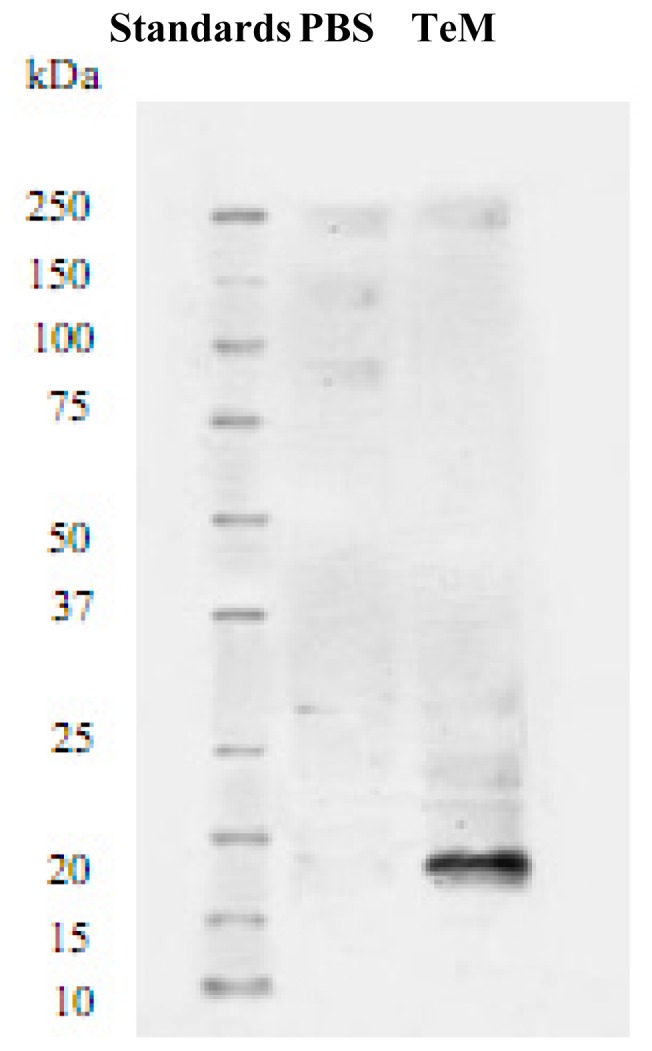
Detection of exogenous TeM protein in retina of 17-day-old mice by western blotting. An immunoreactive band was detected with a molecular mass of approximately 16 kDa in TeM-injected retina, but not in PBS-injected retina.

Exogenous TeM protein was detected by western blot analysis as a prominent band of approximately 16 kDa (which is its expected size) in extracts from P17 TeM-injected retinas. This demonstrates the presence of TeM used in the experiments. In contrast, no immunoreactive band was detected in extracts from PBS-injected control retinas (Figure 4). This demonstrated that the OIR model was successful, and that the exogenous TeM was incorporated into the retina.

## 3. Experimental Section

### 3.1. Preparation of Tenomodulin

TeM (2 mg/mL) was generously provided by Dr. K. Yamana, Teijin Pharmaceutical Co., Ltd. (Hino, Tokyo, Japan). It was dissolved in a solution containing 25 mM HEPES and 0.15 M NaCl at pH 8.3, and stored at −80 °C. The TeM protein has a strong hydrophobic nature, high concentrations that result in precipitation, and freeze-thaw cycles can be repeated 2 to 3 times. The activity of the TeM protein can be maintained for 2 to 3 days in aqueous solution.

### 3.2. Animal Model and Treatment

This study adhered to the ARVO Statement for the Use of Animals in Ophthalmic and Vision Research. Neonatal mice (C57BL/6) were provided by Charles River Laboratories, Osaka, Japan and housed in the animal facility of the Osaka University Graduate School of Medicine. C57BL/6 mice were randomly divided into three groups: a normoxia group (control group), an oxygen-exposed group (OIR group), and a TeM group; each group had one nursing mother and five pups. The mice in the normoxia group were maintained in room air from birth until postnatal day 17 (P17). The mice in the oxygen-exposed group were exposed to 75% ± 2% oxygen (hyperoxia) at postnatal day 7 (P7) for five days and then returned to room air for another five days, producing relatively hypoxic conditions. In the TeM experimental group, 1 μg (0.5 μL) TeM was injected into the vitreous body at P7 with a 32 gauge needle (Hamilton, Reno, NV, USA) in one eye, while the same volume of PBS was injected into the other eye as a control. As soon as the injections were finished, the mice were then exposed to a 75% ± 2% oxygen environment.

### 3.3. Angiography Using High Molecular Weight Fluorescein-Dextran for Retinal Flat-Mounting

Fluorescein angiography of the retina was performed as previously described [29]. On P17, mice were deeply anesthetized by isoflurane (Abbott, Japan) inhalation, and then perfused through the left ventricle with 1 mL of PBS containing 50 mg of 2 × 10^6^ molecular weight fluorescein-dextran (Sigma, St. Louis, MO, USA). Eyes from the normoxia, OIR, and intravitreal injection groups were enucleated and placed in 10% formalin neutral buffer for 4 to 24 h. Corneas and lenses were removed; peripheral retinas were cut and then flat-mounted on a glass slide with glycerol-gelatin. The flat-mounted retinas were viewed by fluorescence microscopy (Leica Microsystems, MZ16F, Japan) and photographed. The central nonperfused area of the retina was quantified from the digital images in a masked fashion, using image processing and analysis software (NIH ImageJ Version 1.61, available in the public domain via the National Institute of Health, Bethesda, MD, USA).

### 3.4. HE Staining

Mice from all groups were sacrificed on P17. Eyes were removed and fixed with 4% paraformaldehyde in PBS for 4 h followed by immersion in 20% sucrose in PBS overnight for frozen sections. The tissues were embedded in optimal cutting temperature (OCT) compound (Tissue-Tek; Sakura Finetechnical Co., Ltd., Tokyo, Japan), frozen at −80 °C, and sectioned at an 8-μm thickness with a cryostat at −20 °C. The sections were taken at roughly 100 μm intervals, spanning the entire retina. Eight-micrometer thick frozen tissue sections adjacent to those used to quantitate retinal neovascularization were stained with Mayer’s hematoxylin (5 min) and eosin (3 min). Specimens were then dehydrated and cover-slipped. Photographs were taken to quantify the neovascular response. Eyes of 5–8 mice from each group were analyzed (each experiment was performed at least two times; the number of animals used for each group was more than 10). For each eye, three sections were randomly chosen and evaluated, neovascular cell nuclei protruding into the vitreous from the ILM were counted using a masked protocol, and the mean number of neovascular nuclei per section was determined.

### 3.5. Immunohistochemistry

Mice from all groups were sacrificed on P17. Eyes were removed and fixed with 4% paraformaldehyde in PBS for 4 h followed by immersion in 20% sucrose in PBS overnight for frozen sections. The tissues were embedded in OCT compound (Tissue-Tek; Sakura Finetechnical Co., Ltd., Tokyo, Japan), frozen at −80 °C, and sectioned at an 8-μm thickness with a cryostat at −20 °C. Sections were obtained at roughly 100 μm intervals, spanning the entire retina. After three 5-min washes with PBS, sections were incubated for 1 h with 5% normal donkey serum at room temperature, and were then incubated overnight at 4 °C with affinity purified goat polyclonal anti-mouse IgG (PECAM-1, M-20, also referred to as CD31; Santa Cruz Biotechnology, Santa Cruz, CA, USA) as the primary antibody, diluted 1:50 with 1% normal donkey serum in PBST (0.3% Triton X in PBS). For the control, normal goat IgG (Santa Cruz Biotechnology, Santa Cruz, CA, USA) was used as the primary antibody. Sections were rinsed in PBS 3 times (5 min each time) and incubated with a secondary Alexa 488 donkey anti-goat IgG-conjugated antibody (Molecular Probes, Invitrogen) plus DAPI (Sigma, St. Louis, MO, USA) for double-staining for 1 h at room temperature. After rinsing the sections again, they were mounted with glycerol-gelatin, viewed under a fluorescence microscope (Axiovert 200; Carl Zeiss, Tokyo, Japan), and photographed.

### 3.6. Western Blot Analysis

Intravitreal-injected retinas from P17 mice were dissected and lysed in RIPA buffer (Sigma, St. Louis, MO, USA) with proteinase inhibitor on ice for gel analysis. Samples were homogenized and then centrifuged at 1.5 K rpm for 5 min at 4 °C. Supernatant was loaded and electrophoresed on 12.5% SDS-poly-polyacrylamide gel and transferred to Hybond-P membranes (Amersham Biosciences, Piscataway, NJ, USA). After preincubation with blocking buffer (5% BSA in PBST) at 4 °C overnight, the membrane was incubated with anti-FLAG M2 monoclonal antibody (Sigma-Aldrich, St. Louis, MO, USA) for 30 min at room temperature. After washing, HRP-conjugated rabbit anti-mouse IgG (Amersham Biotech, Piscataway, NJ, USA) was added for 30 min at room temperature with shaking, according to the manufacturer’s protocol. Visualization was achieved using DAB substrate buffer and DAB chromogen (Dako North America, Inc., Carpinteria, CA, USA).

### 3.7. Statistical Analysis

Each experiment was performed at least two times, and the data were analyzed statistically using Statistical Package for the Social Sciences (SPSS 13.0), using one-way analysis of variance (ANOVA), followed by Scheffé’s pairwise comparison. *p* values of <0.05 were considered statistically significant.

## 4. Discussion

Tenomodulin is a member of a new family of type II transmembrane glycoproteins [25,26]. It is predominantly expressed in dense connective tissues including tendons, ligaments, and eyes; it has been reported that a novel gene encoding TeM has homology to chondromodulin I (ChM-I) [21,23,24]. ChM-I is a 25 kDa glycoprotein that is expressed in the avascular cartilage and at lower levels in the eye and thymus, and it has been previously identified as a tissue-specific inhibitor of angiogenesis in fetal bovine cartilage [27]. TeM has significant amino acid sequence homology (36%) with the ChM-I precursor [21,23]. Cloning of the full-length cDNA of TeM revealed that the TeM protein (317 amino acid residues) has a single transmembrane domain in the *N*-terminal region, 2 N-linked glycosylation sites, and a cysteine-rich domain (Phe225-Val317) in the *C*-terminal region [21,23,24]. TeM and ChM-I exhibit the strongest sequence identity (65%) within the 63-amino-acid *C*-terminal region, which corresponds to the predicted anti-angiogenic functional domain of ChM-I (Phe272-Val334) [23]. Both TeM and ChM-I carry 8 cysteine residues in their *C*-terminal domains, and the spacing of the 8 cysteine residues in TeM is almost identical with that in ChM-I [21,23]. However, in contrast to ChM-I, the mature form of which is a secreted protein existing in the extracellular matrix after being processed from a transmembrane-type precursor [21], the TeM protein is presumably located at the cell surface, and it also lacks the hormone-processing signal present in the ChM-I precursor [21,23]. Because of the similarity in the *C*-terminal characteristics between TeM and ChM-I, and the structural analogy noted between skeletal connective tissues and the eye in terms of hypovascularity and the components of their extracellular matrix, it is possible that TeM also participates in the maintenance of avascular conditions in the eye.

Both TeM and ChM-I have been reported to inhibit proliferation and tube morphogenesis of vascular endothelial cells *in vitro* and have a strong anti-tumor effect *in vivo*. Clinical and laboratory studies have also reported robust evidence indicating that tumor angiogenesis is inhibited by administration of anti-angiogenic factors. Oshima *et al.* have also reported that recombinant ChM-I protein effectively suppresses the growth of chondrosarcoma and colon adenocarcinoma by inhibiting angiogenesis *in vivo*[26]. It is likely, therefore, that the *C*-terminal ChM-I-like domain of TeM has anti-tumor activity *in vivo*, and indeed expression of both Ad-shTeM (the 116 amino acids of the human TeM *C*-terminal) and Ad-shChM-I (the 120 amino acids of the human ChM-I precursor *C*-terminal) result in the effective inhibition of angiogenesis, followed by suppression of tumor growth [20,23]. The conserved anti-angiogenic domain present in both TeM and ChM-I might therefore be a useful target for tumor treatment, although the mechanisms underlying TeM or ChM-I activity are not fully understood.

The anti-angiogenic potential of TeM on retinal neovascularization *in vivo* also remains unclear. Previously, Docheva *et al.* reported that mice lacking both TeM and ChM-I showed normal OIR retinal vasculature, and that double-deficient mice did not have changes in tendon vessel density. This suggested that TeM is a regulator of tenocyte proliferation and is involved in collagen fibril maturation, but did not support a role for TeM in angiogenesis *in vivo*[25]. The controversial *in vitro* and *in vivo* studies regarding the role of ChM-I and TeM in angiogenesis led us to investigate TeM function in retinal neovascularization, which had not been studied in oxygen-induced retinopathy (OIR) in C57BL/6 mice. In the present study, we used a highly reproducible mouse model of ischemia-induced retinopathy [29]. This mouse model of OIR is useful in assessing medical intervention on proliferative retinal vasculopathies [29]. As demonstrated in this report, exogenous TeM protein was successfully detected by western blotting as a 16 kDa immunoreactive band in retinal extracts from TeM-injected mice. This represents the expected molecular size of a TeM fragment, indicating that this fragment of TeM was used in this study, since previous studies have reported the molecular mass of the full-length TeM protein to be approximately 45 kDa [20,25,26]. We found that this fragment of TeM is antiangiogenic and prophylactic injection of the fragment partially prevents ischemia-induced proliferative retinopathy. The molecular mass of this fragment corresponds to a *C*-terminal fragment of TeM after cleavage at a predicted site [25], and the *C*-terminal of full-length TeM is exposed on the cell surface [26]. Furthermore, the *C*-terminal domain of both TeM and ChM-I exhibits antiangiogenic properties [26]; thus, it can be speculated that the full-length TeM protein is also antiangiogenic. Regardless of the interpretation of these results, this is an interesting finding, and further research and confirmation is necessary.

The mechanism of inhibition of pathological angiogenesis by TeM *in vivo* remains unclear. Previous studies, both *in vitro* and *in vivo*, have demonstrated that VEGF plays a major role in stimulating retinal neovascularization [6,8,30–34]. VEGF levels have been found to be elevated in the retina and vitreous cavity of patients with ischemic ocular neovascular disorders, as well as in animal models of ocular neovascularization [3,4,8,35]. Many studies have shown that blocking VEGF activity can prevent the development of retinal neovascularization [31,35–37]. Thus, we suspect that prior injection of TeM inhibits VEGF activity that is stimulated by moving the mice to the relatively hypoxic conditions of normal room air. Previous studies have proposed that upregulation of VEGF in the aqueous humor and vitreous body plays an important role in intraocular angiogenesis [20,35,38]. In a physiological state, expression and localization of VEGF are also detectable in normal vascularized ocular tissues, such as the conjunctiva, iris, retina, and choroid-RPE complex [21,39]. These features suggest the presence of antiangiogenic agents in the avascular parts of the eye, such as the sclerocornea, aqueous humor, and vitreous body, for the prevention of vascular invasion. It is of interest that TeM and other inhibitory factors such as PEDF are constitutively present in the aqueous humor and vitreous body at a high concentration, possibly working to counterbalance the angiogenic stimulators [15,20,28]. It has also been concluded by others that the retina is the major tissue where TeM and ChM- I are coexpressed [20]. These angiogenic characteristics include the *C* terminus of TeM restricting vascular formation in the retina. The fact that TeM is a transmembrane protein and the presence of its antiangiogenic domain on the cell surface may work as an antiangiogenic barrier for the physiological prevention of vascular invasion into hypovascular tissues [20, 26]. On the basis of these studies, we speculate that the possible mechanism of TeM inhibition of retinal neovascularization in our mouse model maybe as follows: a hypoxic state stimulates a high expression of VEGF; meanwhile, ectogenic TeM administration associated with the endogenous TeM leads to the elevated levels of TeM, thus preventing pathological angiogenesis. A recent study demonstrated that the *C*-terminal fragments of TeM and ChM-I, both of which are located downstream of the transmembrane domain, are responsible for their antiangiogenic activity [20]. This may be another factor contributing to their inhibition of retinal neovascularization.

## 5. Conclusions

The findings of this study indicate that TeM is effective, at least in part, in preventing pathologic angiogenesis in a mouse model of ischemic retinopathy. A better understanding of the biology and mechanism of action of TeM may someday lead to the development of novel and more effective therapeutic approaches for the treatment of pathologic neovascular conditions. Thus, these findings offer preventive and therapeutic potential for the management of pathologic intraocular angiogenesis.
